# Do the fluorescent red eyes of the marine fish *Tripterygion delaisi* stand out? In situ and in vivo measurements at two depths

**DOI:** 10.1002/ece3.4025

**Published:** 2018-04-15

**Authors:** Ulrike K. Harant, Matteo Santon, Pierre‐Paul Bitton, Florian Wehrberger, Thomas Griessler, Melissa G. Meadows, Connor M. Champ, Nico K. Michiels

**Affiliations:** ^1^ Animal Evolutionary Ecology Institute of Evolution and Ecology Department of Biology Faculty of Science University of Tuebingen Tuebingen Germany; ^2^ Present address: Department of Biology Saint Francis University Loretto PA USA

**Keywords:** coloration, Michelson contrast, visual communication, visual contrast, visual signal

## Abstract

Since the discovery of red fluorescence in fish, much effort has been invested to elucidate its potential functions, one of them being signaling. This implies that the combination of red fluorescence and reflection should generate a visible contrast against the background. Here, we present in vivo iris radiance measurements of *Tripterygion delaisi* under natural light conditions at 5 and 20 m depth. We also measured substrate radiance of shaded and exposed foraging sites at those depths. To assess the visual contrast of the red iris against these substrates, we used the receptor noise model for chromatic contrasts and Michelson contrast for achromatic calculations. At 20 m depth, *T. delaisi* iris radiance generated strong achromatic contrasts against substrate radiance, regardless of exposure, and despite substrate fluorescence. Given that downwelling light above 600 nm is negligible at this depth, we can attribute this effect to iris fluorescence. Contrasts were weaker in 5 m. Yet, the pooled radiance caused by red reflection and fluorescence still exceeded substrate radiance for all substrates under shaded conditions and all but *Jania rubens* and *Padina pavonia* under exposed conditions. Due to the negative effects of anesthesia on iris fluorescence, these estimates are conservative. We conclude that the requirements to create visual brightness contrasts are fulfilled for a wide range of conditions in the natural environment of *T. delaisi*.

## 1 INTRODUCTION

The characteristics of downwelling light change rapidly with depth in the water column, from directional, bright, and spectrally broad near the surface to scattered, dim, and spectrally narrow at depth (Jerlov, 1968; Loew & Zhang, 2006; Lythgoe, 1979; Marshall et al., 2003). The two main underlying processes responsible for these changes are light absorption and scattering (Jerlov, 1968; Loew & Zhang, 2006; Lythgoe, 1979; Marshall et al., 2003). Light absorption is particularly strong for longer wavelengths, resulting in a skew toward intermediate, blue‐green wavelengths in the visible spectrum. The remaining light is increasingly scattered as it penetrates into the water column resulting in soft, homogeneous lighting that lacks sharp illumination boundaries. These effects have profound consequences for animal coloration as well as visual perception. In shallow water, the ambient spectrum exceeds the spectral sensitivity range of most fish at both ends of the visible spectrum, the euryspectral zone (Meadows et al., 2014). With increasing depth, the range of wavelengths available in the ambient light quickly narrows. Around 20 m depth, the euryspectral zone gradually changes into the stenospectral zone, where the spectral sensitivity of fish is broader than that of the available ambient light (Meadows et al., 2014).

Most natural colors originate from wavelength‐specific absorption by pigments, or through structural mechanisms. In nonfluorescent pigments, possible hues are therefore strictly limited by the availability of specific wavelengths in the ambient spectrum. Fluorescent pigments do not have this limitation, provided that the right excitation wavelengths are available. They transform absorbed photons of a given range of wavelength (e.g., in the blue‐green range) and re‐emit light at longer, less energetic, wavelengths (e.g., yellow or red). Although fluorescent pigments are widespread in benthic marine organisms (Alieva et al., 2008; Eyal et al., 2015; Marshall & Johnsen, 2017; Sparks et al., 2014), their presence in fish living in shallow water (0–40 m) has only recently been confirmed (Anthes et al., 2016; Gerlach et al., 2016; Michiels et al., 2008; Sparks et al., 2014). To date, several studies investigated potential functions of red fluorescence in fish, including intraspecific communication, camouflage, and prey detection (Anthes et al., 2016; Detecting the Detector; Harant & Michiels, 2017; Meadows et al., 2014). In this study, however, we only focus on assessing whether such a red signal stands out in front of natural backgrounds and thus generates a perceptible contrast.

**Figure 1:**
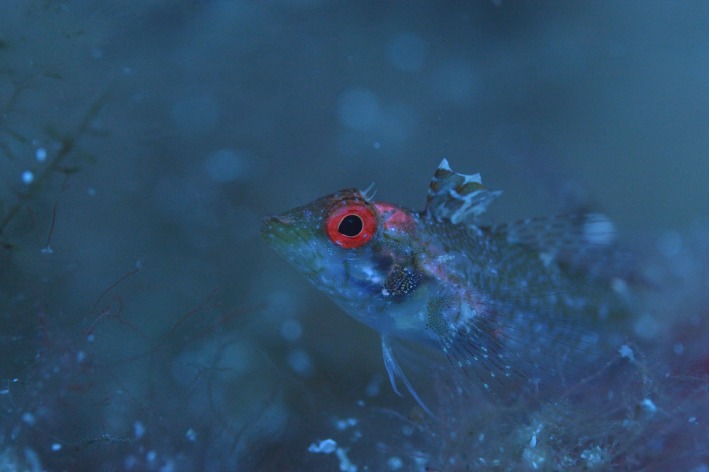
*Tripterygion delaisi* displaying its conspicuous red iris fluorescence at 30 m depth. Picture taken with Nikon D4, LEE 287 Double C. T. Orange filter, and manual white balance, without postprocessing (Nico K. Michiels). Note that LEE 287 is not a long‐pass filter (as is, e.g., LEE 105 Orange or LEE 106 Primary Red). It is used to correct a bluish cold‐white scene to a warmer spectrum in photography (C. T. = “Correct to Tungsten”). Combined with Manual White Balance, this results in pictures that show colors at depth, including fluorescence, as perceived by a human diver

The black‐faced triplefin *Tripterygion delaisi* possesses remarkably red fluorescent irides. Its fluorescence is among the strongest of all fish measured thus far (Anthes et al., 2016) and can be perceived by the human eye without the aid of an excitation source or the use of long‐pass viewing filters (Figure 1). Yet, its fluorescence appears still weak relative to the ambient light. However, a recent study showed that this weak fluorescent signal can generate a chromatic and achromatic contrast between iris radiance and the background radiance that is strong enough to be perceived by conspecifics, at least for neutral, nonfluorescent backgrounds (Bitton et al., 2017).

Given that natural backgrounds are very diverse, and often fluoresce in the red waveband, we scrutinize the visual model empirically by directly measuring whether iridal radiance in *T. delaisi* generates perceptible contrasts with the background radiance from the natural substrates. To this end, we characterized the natural light environment of *T. delaisi* by measuring the down‐ and sidewelling light field as well as the radiance of typical substrates under euryspectral (5 m) and stenospectral conditions (20 m). *T. delaisi* uses shaded as well as exposed parts of its home range for foraging, which was also considered in the choice of sites. Furthermore, we measured iris radiance in anesthetized *T. delaisi* in situ under these conditions. Contrast estimates of substrate and iris radiance allowed us to identify combinations of substrate, depth, and exposure under which iris radiance stands out against the background from the perspective of the visual system of the fish.

## 2 MATERIALS AND METHODS

The black‐faced triplefin *Tripterygion delaisi* is a small, benthic fish which lives in rocky habitats between 5 and 50 m depth along the Mediterranean and eastern Atlantic coasts (Louisy, 2002). It feeds mainly on small, benthic invertebrates (Zander & Hagemann, 1989; Zander & Heymer, 1970). Except for the breeding season, when males develop prominent coloration, individuals are highly cryptic against their natural background, with no obvious sexual dimorphism. *Tripterygion delaisi* displays highly fluorescent irides with an average peak emission (λ_max_) of 609 nm with a full width at half maximum range of 572 nm to 686 nm (Bitton et al., 2017). Furthermore, it can perceive its own red fluorescence (Bitton et al., 2017; Kalb, Schneider, Sprenger, & Michiels, 2015), which is regulated from nearly absent to maximum brightness through melanosome dispersal or aggregation in melanophores in less than 30 s (Wucherer & Michiels, 2014).

### 2.1 Field site

Field data were collected while scuba diving at three sites at the Station de Recherches Sous‐marines et Océanographiques (STARESO) Calvi, Corsica, France, in June–July 2014 and 2015. The shallow site (1) is adjacent to STARESO and characterized by rocky slopes, steep walls, and granite boulders down to 12 m. Exposed hard substrates are covered with a diverse community of green, red, and brown algae (Appendix S1). Shaded parts are dominated by coralline red algae and sedentary animals (sponges, cnidarians, bryozoans, ascidians). Flat sandy sediments start at the bottom of the slope and are covered with seagrass (*Posidonia oceanica*), leaving only small patches of rubble and sand. The seagrass meadow slopes gently into deeper water, down to more than 30 m depth. The deep site (2) is located 1 km East of STARESO (“La Bibliothèque”). It features large granite boulders of 1–6 m across from above the surface down to 25 m. A seagrass meadow starts at the bottom of the slope. Areas between the boulders are covered with rubble and sand. The boulders are vegetated mainly by algae including calcareous algae, and some sponges and ascidians, particularly in the permanently shaded parts.

### 2.2 General spectroradiometric setup

**Figure 2:**
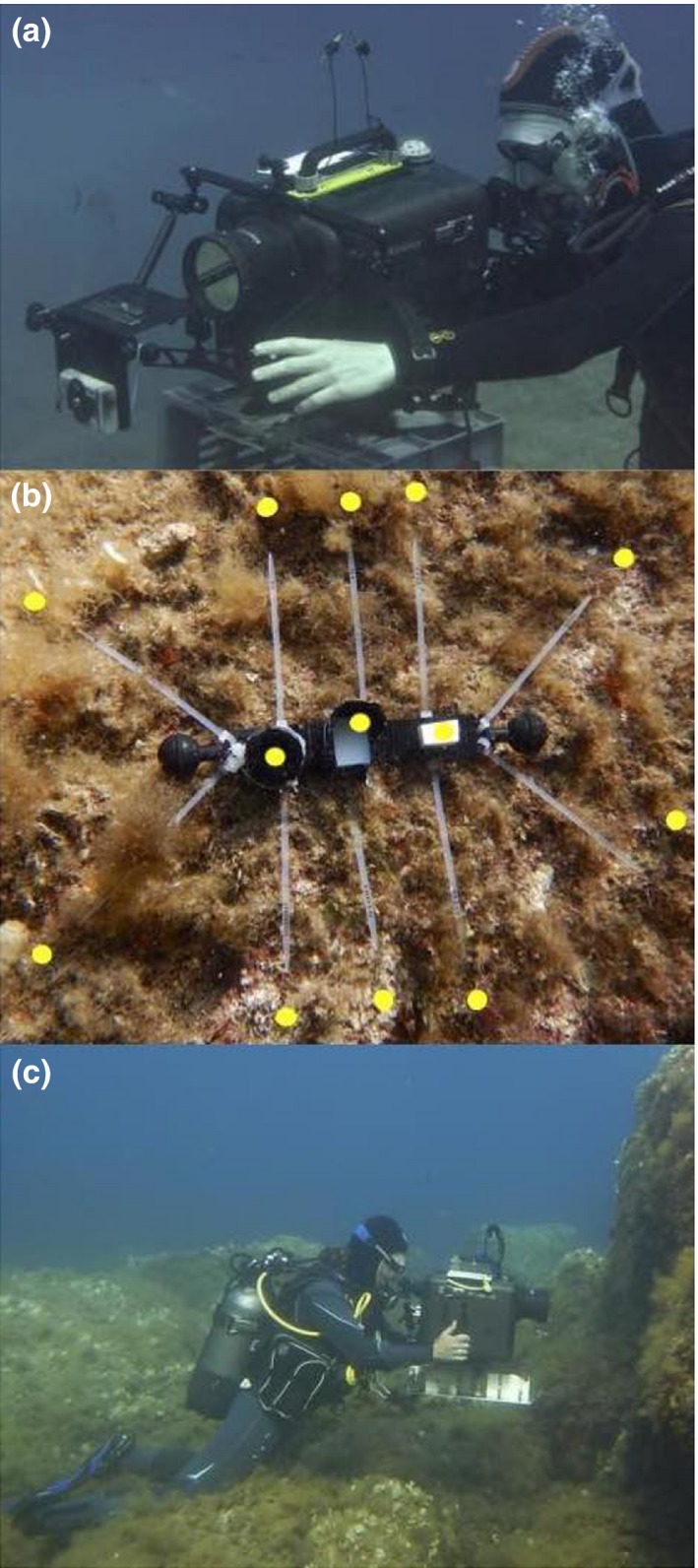
(a) Iris radiance measurements taken with a radiospectrometer aiming at a secured, slightly anesthetised fish at 20 m depth. (b) Substrate transect device with reflectance standards in the center (left to right): black standard, shaded diffuse white standard (PTFE) and exposed diffuse white standard (PTFE). The latter was used for the calculations presented here. Spectral measurements were taken approx. 1 cm above each of the 10 cable binder tips (yellow spot) while pointing horizontally at the substrate. The length of the central black carrier is 22.5 cm. (c) Substrate radiance measurements were taken at 5 and 20 m depth using a calibrated spectroradiometer in a custom‐made underwater housing (BS Kinetics)

Radiance measurements were taken with a calibrated PhotoResearch SpectraScan PR‐740 spectroradiometer in a custom‐made underwater housing (BS Kinetics) with a calibrated MS‐75 standard lens (Figure 2 a, c). The PR‐740 is an all‐in‐one aim‐and‐shoot spectrometer with Pritchard optics. It allows to visually focus on a target from a distance with set acceptance angles between 0.1° and 1° and measures absolute radiance (watts · sr^− 1^ · m^−2^ · nm^−1^) in the 380–780 nm range with a 1‐nm resolution using a bandwidth of 8 nm. Due to its cooled sensor, this spectroradiometer captures even very weak signals with little noise at short exposure times. A compass, a level indicator, and an electronic depth gauge were mounted on top of the housing for accurate positioning. Measured radiances were subsequently corrected for the transmission of the port of the underwater housing and transformed into photon radiance (photons · s^−1^ · sr^−1^ · m^−2^ · nm^−1^) by multiplication with *wavelength* · 5.05 · 10^15^ at each wavelength (Johnsen, 2012).

### 2.3 Radiance of substrates frequented by *T. delaisi*

We collected spectral measurements throughout the day (07:30–18:00) from 29 typical *T. delaisi* substrates that were either exposed or shaded at 5 and 20 m depth. We defined a substrate to be shaded if it was permanently shaded by, for example, overhanging rocks. Compass direction and surface slope were chosen to cover representative variation. However, the effect of compass direction was negligible compared to substrate exposure (shaded/exposed) and time of day. We therefore omitted orientation from the results. Note that very steep, vertical, or overhanging surfaces could not be measured due to handling limitations of the underwater housing, although these areas are also frequented by *T. delaisi*.

To standardize measurements and assess small‐scale variation of microhabitat characteristics, a small transect device was created (Figure 2b). It defined 10 arbitrary measurement points peripherally located around three centrally positioned standards: an exposed polytetrafluoroethylene (PTFE) diffuse white reflectance standard (Berghof Fluoroplastic Technology GmbH), as a combined measure of downwelling and sidewelling light, a shaded PTFE standard to assess sidewelling light only (not used in this study), and a black standard (dark opening of a small vial covered with black cloth inside and outside) as a proxy for the amount of scattered light between spectroradiometer and substrate. However, the signal of the black standard was mostly too weak to be measured and was therefore not considered for later calculations. At each station, we first measured the three standards, followed by 10 locations on the substrate, 1 cm above each tip of the 10 measurement markers (Figure 2b), followed by a second measurement of each standard. Standards were measured twice to ensure that the overall light environment remained stable during a transect measurement. In each transect, all measurements were repeated three times, including the standards and the 10 substrate spots. All measurements were taken from a fixed distance of 60 cm, the minimal focal distance of the spectroradiometer in the submerged housing.

To assess whether substrate radiance exceeded the radiance of the exposed diffuse white standard (DWS) in some parts of the spectrum, we first averaged measurements for each substrate type within a transect. We then calculated relative radiance at each nanometer as the radiance of that specific substrate type relative to the exposed DWS of that transect. At each wavelength, values are expected to be generally smaller than 1, unless when the substrate featured a combination of reflectance and fluorescence that lead to greater radiance than the ambient light. Note that we use the term “relative radiance” rather than the more common term “reflectance” because of the combined effects of reflection, transmission (if any), and fluorescence in our radiance measurements. All raw and derived substrate measurements are provided in Appendices S2 and S3.

### 2.4 Iris measurements of *T. delaisi*

Iris radiance was measured at 5 m (site 1, *n *=* *16 individuals) and 20 m depth (site 2, *n *=* *18 individuals) using the same spectroradiometric setup as described above but with an added SL‐0.5× macro lens. In addition, we equipped the spectroradiometer with a LEE 287 Double C.T. Orange filter, which reduces the abundant blue‐green light, allowing longer exposure times to capture better readings in the weak red waveband. We corrected our measurements for filter transmittance when processing the data (see below). A collection team first caught fish with hand nets at the target depth and brought them to the nearby measurement location in 50 ml Falcon^™^ tubes. The measurement team then anesthetized fish with diluted clove oil and gently placed them in a transparent plastic holder fixed to a small table attached to the front of the housing port (Figure 2a). The whole head of the fish was fully exposed to the ambient light. Fish were measured with the measured eye facing south (sun‐exposed, more directional light) or north (shaded from direct sunlight, more scattered light). Instead of the PTFE diffuse white reflectance standard, we used white waterproof paper (Avery Zweckform) as a diffuse white standard (see Appendix S4 for comparative measurements). Measurements of the white standard were followed by four fixed positions on the fluorescent iris (top, right, bottom, and left). The measurement dot (shown as a small black disk in the viewfinder) was always smaller than the width of the iris. Each series ended with an additional measurement of the white standard. Upon completing one eye, the dive buddy turned the fish around and the second eye was measured. All data were transformed to photon radiance and corrected for reflectance (waterproof paper relative to PFTE, Appendix S2), equipment transmission, and the orange filter. The measurements taken at the four positions on each eye were averaged per individual to reduce variation. Similar to the substrate measurements, we also transformed iris radiance to relative iris radiance. All raw and relative radiance measurements are provided in Appendix S5.

### 2.5 Anesthesia effect

To assess the effect of anesthesia on iris radiance in *T. delaisi*, we measured 10 freshly caught individuals originating from 5 and 20 m depth (*N *=* *20) in the field (STARESO, Calvi, France) and measured iris radiance before and after treating them with clove oil. We used the same spectroradiometric setup and procedure as described in Harant et al. (2016) with two blue Hartenberger Mini Compact LCD dive torches (7 × 3.5 W 450 nm bulbs) with an additional short‐pass filter (Thorlabs FD2C subtractive dichroic color short‐pass filter) serving as light source. Before adding a fish to the measurement chamber, we took three measurements of a nonfluorescent red diffuse reflectance standard (Labsphere SCS‐RD‐010) to check for stray red light and to ensure constancy of the light conditions in the room. Afterwards, three measurements of each individual were made before and after applying the clove oil treatment. To avoid oil droplets in the measurement chamber, fish anesthesia took place in a separate anesthesia bath (1‐L plastic cubs). Fish remained in the anesthesia bath until they lost their balance but still showed continuous operculum movement. Afterwards, each fish was rinsed with fresh sea water and immediately transferred back to the measurement chamber for further spectroradiometric measurements. Data were corrected for the used filter (Lee Filters, Double C.T. Orange 287) and converted from watts · sr^− 1^ · m^−2^ · nm^−1^ into photon radiance (photons · s^−1^ · sr^−1^ · m^−2^ · nm^−1^) by multiplication with *wavelength* · 5.05 · 10^15^ at each wavelength (Johnsen, 2012). We then calculated a spline predictor for iris photon radiance per capture depth and treatment. A spline predictor ratio per wavelength was then calculated between treatments (separately for 5 and 20 m depth) and multiplied with the according mean iris radiance to correct for the anesthesia effect.

### 2.6 Data analysis

To compare iris radiance against substrate radiance, we calculated chromatic contrasts using the receptor noise model described by Vorobyev and Osorio (1998) and achromatic contrasts using Michelson contrasts (Michelson, 1995). The receptor noise model was parameterized using species‐specific visual characteristics (Bitton et al., 2017; Fritsch, Michiels, & Collin, 2017). In short, we produced photoreceptor sensitivity curves based on a vertebrate template (Govardovskii et al., 2000) using peak sensitivities at 450, 517, and 530 nm for the short‐, medium‐, and long‐wavelength‐sensitive photoreceptors, respectively, and used the ocular media transmission properties described in Bitton et al. (2017). We set the Weber fraction at 0.05 as suggested for other teleosts (e.g., Wilkins, Marshall, Johnsen, & Osorio (2016), and relative photoreceptor densities of 1:4:4 (short‐, medium‐, long‐wavelength‐sensitive photopigments) as is found in the triplefin fovea (Fritsch et al., 2017). The chromatic contrast values generated by this model are in just‐noticeable differences (JNDs), with scores above 1.0, indicating that the colors are distinguishable from one another. We calculated the quantum catches of the mean iris (*Q*_i_) and mean substrate radiance (*Q*_s_) captured by the double cones (medium‐ and long‐wavelength photoreceptors) and determined the achromatic Michelson contrasts as follows (Michelson, 1995):

$$C=\frac{\left(Q_{i}-Q_{s}\right)}{\left(Q_{i}+Q_{s}\right)}$$

where *C* indicates whether iris radiance was stronger (0 < *C *≤* *1) or weaker (−1 ≤ *C *<* *0) than substrate radiance. Whether a contrast is detectable for fish depends on several factors including the overall brightness in the environment, the size of the stimulus as well as the distance to the stimulus (Cronin, Johnsen, Marshall, & Warrant, 2014). However, in the euphotic zone, fish with relatively well‐developed eyes looking at a stimulus roughly matching their eye size within an ecologically relevant distance have a contrast threshold of 1–2% under bright light conditions (Cronin et al., 2014). Hence, under optimal daylight conditions, it is assumed that a Michelson contrast near *C = *0.018 should be detectable by most fish (Anthony, 1981; Douglas & Djamgoz, 2012; Hawryshyn, Arnold, Chaisson, & Martin, 1989; Hester, 1968). We performed Welch's t tests comparing the mean radiance of the iris to that of various substrates in all conditions, only for substrates with 10 or more measurements. Michelson contrast differences were considered significant if greater than the absolute value of 0.018. We controlled for false discovery rates by adjusting the *p*‐value for multiple comparisons following Benjamini and Yekutieli (Benjamini & Yekutieli, 2001) using the “p.adjust” function in R. Visual models were performed using the R package “pavo” (Maia et al., 2013).

## 3 RESULTS

### 3.1 Relative radiance of substrates

**Figure 3:**
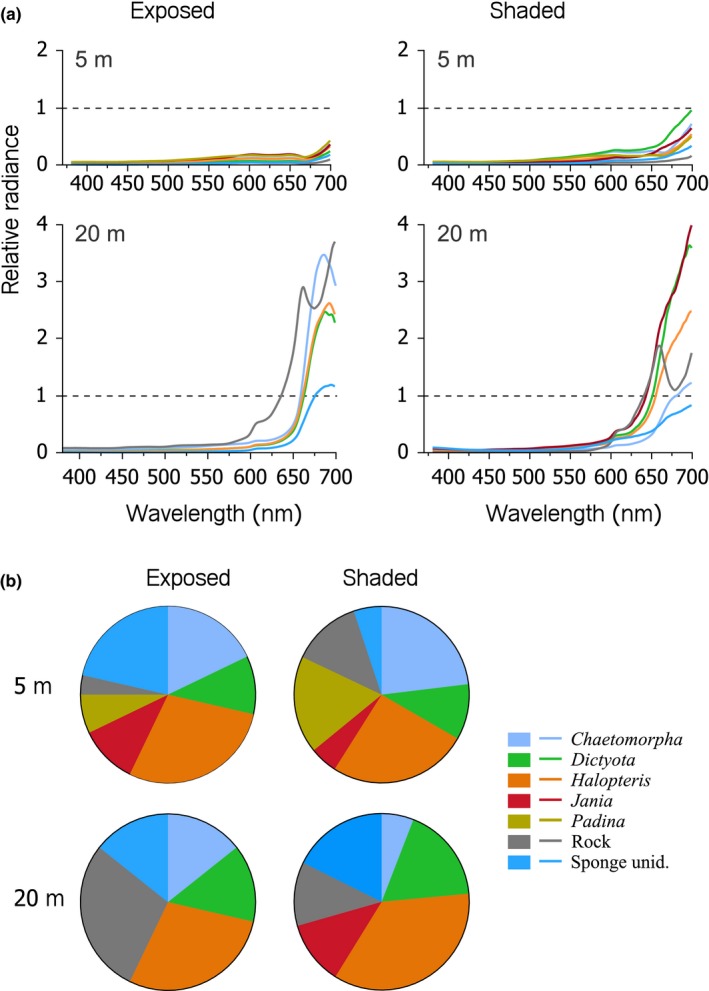
(a) Line plots showing mean relative radiance (prop.) of typical *Tripterygion delaisi* substrate types as a function of wavelength at 5 and 20 m depth (rows) under sun‐exposed and shaded conditions (columns). Values exceeding 1 (black dashed line, referring to the radiance of the exposed diffuse white standard) indicate substrates that emitted more light at a specific wavelength than was available in the light spectrum, a typical signature of strong fluorescence. (b) Pie charts showing relative abundance of substrate types measured at each combination of depth and exposure. For a detailed species list, see Appendix S1

At 5 m, substrate relative radiance was largely below one, indicating that fluorescent components in the substrate were too weak to compete with the ambient light (Figure 3). At 20 m, however, substrate relative radiance substantially increased at longer wavelengths between 600 and 700 nm. This effect can be attributed to fluorescence from photosynthetically active organisms. Depending on substrate type and exposure, substrate radiance exceeded that of ambient light (indicated by the line at *y *=* *1 in Figure 3) by a factor of up to four in the 600–700 nm range.

### 3.2 Relative radiance of *Tripterygion delaisi* irides

**Figure 4:**
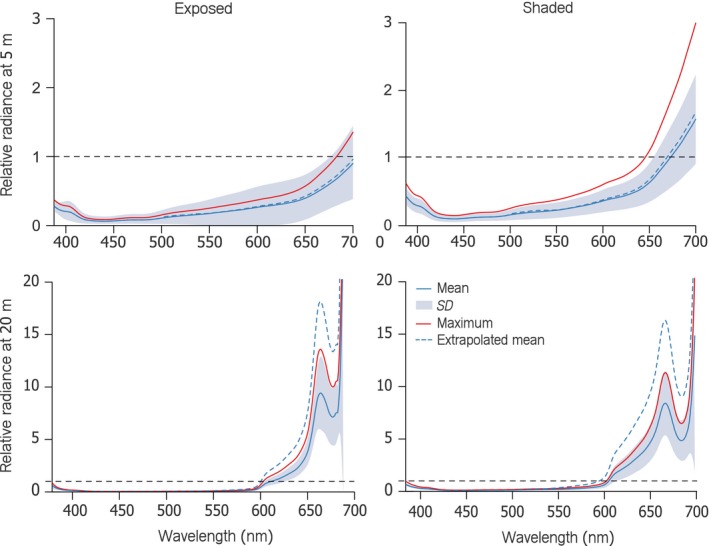
Line plot showing iris relative radiance (prop.) of *Tripterygion delaisi* as a function of wavelength under exposed (left column) and shaded (right column) conditions at either 5 m (upper row) or 20 m depth (lower row). Blue lines represent means ± SD (shading) of all fish. Red lines indicate the maximum relative radiance averaged across individuals (*n *=* *34). Values exceeding 1 (horizontal black dashed line) indicate that more photons were emitted by the fish iris at that wavelength than were available in the ambient spectrum, indicative of red fluorescence (assuming absence of specular reflection). The blue dashed curve shows the estimated brightness of the iris without clove oil anesthesia (see Methods)

At 5 m, relative radiance of fish irides exceeded 1 in the deep red range (>680 nm) under shaded conditions (eye facing North) only (Figure 4). This can be explained by the strong red component in the down‐ and sidewelling light that overrides the relative radiance signal in exposed fish (eye facing South). At 20 m, however, iris radiance exceeded diffuse white standard radiance by up to nine times (one single measurement), irrespective of exposure—an effect that can only be attributed to iris fluorescence.

Using clove oil for anesthesia leads to a noticeable reduction in iris radiance (Wucherer & Michiels, 2014). This is especially true for fish from 20 m depth, where anesthesia decreases iris radiance by 46% on average compared with nonanesthetized fish. Fish caught at 5 m depth reduced their iris radiance by only 14% on average after being anesthetized. The depth dependency can be explained by reduced iridal melanophore densities in individuals at greater depths (Harant et al., 2016; Wucherer & Michiels, 2014). All measurements presented here are therefore underestimating natural iris radiance, particularly in individuals from deeper water. We therefore added an “extrapolated mean” as an estimate for nonanesthetized individuals in Figure 4.

### 3.3 Chromatic vs. achromatic contrast

**Figure 5:**
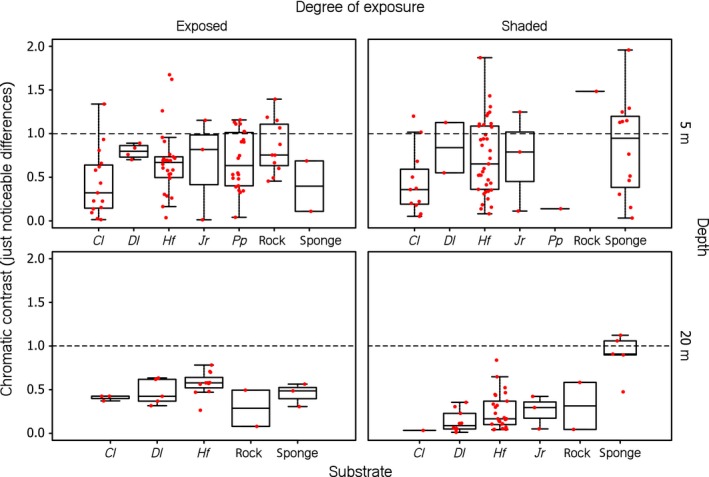
Boxplots of chromatic contrasts between mean iris and mean substrate radiance per substrate type. Data points (in red) represent processed, individual measurements. The horizontal line in the graph indicates the threshold of color discrimination, set at 1 just‐noticeable difference (JND). Values above 1 indicate that a contrast is likely to be perceived by *Tripterygion delaisi*. The algal species covering the substrate are coded as follows: *Cl, Chaetomorpha linum*;* Dl, Dictyota linearis*;* Hf, Halopteris filicina; Jr, Jania rubens*; and *Pf, Padina pavonia* (see Appendix S1)

**Figure 6:**
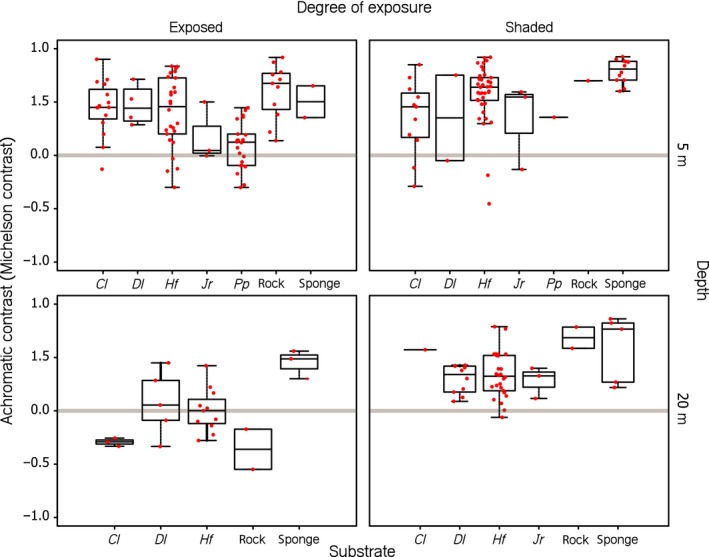
Boxplots showing achromatic contrast (Michelson contrast) between mean iris and mean substrate radiance per substrate type. Data points (in red) represent processed, individual measurements. The achromatic contrasts are unitless (see Methods). Values above or below the gray horizontal strip (*C *=* *0 +/‐ 0.018) indicate that a contrast is likely to be perceived by *Tripterygion delaisi* with positive values indicating irides being brighter than the substrate. The algal species covering the substrate are coded as follows: *Cl, Chaetomorpha linum; Dl, Dictyota linearis; Hf, Halopteris filicina; Jr, Jania rubens;* and *Pf, Padina pavonia* (see Table 1 for statistical significances and Appendix S1)

Under all conditions, chromatic contrasts between the iris radiances and the substrate radiances were under 1 just‐noticeable difference, indicating that triplefins would not be able to detect chromatic differences between the iris radiance of conspecifics and the radiance of the various substrates (Figure 5). However, achromatic contrast values differed significantly from zero under most tested scenarios, with positive Michelson contrasts values, indicating that fluorescing irides are usually brighter than background substrates (Figure 6, Table 1). Under exposed conditions at 5 m depth, iris radiance was greater than substrate radiance for all but *Jania rubens* and *Padina pavonia*, both of which are relative bright algae compared to the other substrates. At the same depth but shaded conditions, however, the iris radiance was also greater than that of the algae *Jania rubens* and *Padina pavonia*, perhaps because the blue‐green dominated sidewelling light field increased fluorescence in the triplefin but not in the substrates. At exposed sites at 20 m, iris radiance generally did not exceed substrate radiance except for sponges. In contrast, in shaded sites at 20 m, the iris radiances were always brighter than that of the substrates, again showing the potential role of fluorescence in increasing the relative brightness of the iris in shaded locations. Note that due to low sample size (*N*) of some substrates, we only provide statistical analyses for substrates with *N *>* *10.

**Table 1:** Summary statistics from Welch's *t* tests comparing the Michelson contrast between the radiance of *Tripterygion delaisi’*s irides to that of common substrates under four environmental light conditions. Tests were only performed for substrates with at least 10 replicate measurements. We controlled for false discovery rates due to multiple comparisons following Benjamini and Yekutieli (Benjamini & Yekutieli, 2001). The algal species covering the substrate are coded as follows: *Cl, Chaetomorpha linum*;* Dl, Dictyota linearis*;* Hf, Halopteris filicina*;* Jr, Jania rubens*; and *Pf, Padina pavonia* (see Appendix S1)

Depth (m)	Degree of exposure	Substrate	*t*	*df*	*p*
5	Exposed	*Cl*	6.32	14	<.001
		*Hf*	5.89	25	<.001
		*Pp*	1.56	21	.14
		Rock	7.41	10	<.001
5	Shaded	*Cl*	3.23	10	<.001
		*Hf*	12.41	36	<.001
		Sponge	23.98	11	<.001
20	Exposed	*Hf*	‐0.20	10	.58
20	Shaded	*Dl*	6.53	9	<.001
		*Hf*	7.55	25	<.001

## 4 DISCUSSION

While iris radiance is unlikely to result in a detectable chromatic contrast when viewed against the natural background, our data suggest that *Tripterygion delaisi* should be able to perceive the resulting achromatic contrast under a broad range of conditions. Except for exposed conditions at 20 m depth, iris radiance almost always exceeded substrate radiance under all tested scenarios, including shallow, euryspectral conditions. However, due to the effect of anesthesia on iris fluorescence, these estimates are conservative. Consequently, our work confirms empirically that iris radiance (reflection + fluorescence) in *T. delaisi* is strong enough to generate visual achromatic contrasts in a large part of its natural environment, particularly under shaded conditions (Cronin et al., 2014; Meadows et al., 2014; Bitton et al. 2017) produced similar results through visual modeling, but assuming an achromatic, nonfluorescent background. Our results now confirm that those results may hold against complex, partly fluorescent backgrounds as well.

The lack of longer wavelengths along with the reduced overall brightness makes stenospectral habitats particularly suitable for the use of fluorescence to generate contrast (Cronin et al., 2014; Meadows et al., 2014). This might explain why some particularly strongly fluorescing species are restricted to deeper water such as several species of *Bryaninops*,* Ctenogobiops*, or *Crenilabrius* (Anthes et al., 2016). However, our data suggest that the achromatic contrast is weak under exposed conditions at 20 m depth compared to other conditions, which might be explained by the low sample size at this depth and exposure.

Although Anthes et al. (2016) did not find a correlation between increasing depth and red fluorescence across species, it is safe to assume that red fluorescence is more likely to contribute to visual signaling in deeper water rather than in shallow water. In fact, when analyzing individuals collected at 5 and 20 m within single species (including *T. delaisi*.), Meadows et al. (2014) found that fluorescence radiance increased with depth within species when measured under identical laboratory conditions.

Although we identified several substrate types on which the red fluorescent iris of *T. delaisi* is particularly likely to generate perceptible achromatic contrasts, we need to emphasize that certain typical microhabitats could not be measured. Due to handling limitations of the underwater housing, and the fact that the transect device could only be used on upward facing substrates (Figure 2b), we could not take measurements from underneath overhangs or in crevices, where triplefins are also frequently found. However, given that these shaded sites are exclusively illuminated by blue‐green, sidewelling light, the achromatic contrast generated by iris radiance against the substrate is expected to be relatively high, except in locations where encrusting red calcareous algae are common. The latter substrate often covers large areas inside crevices and exhibits very strong red fluorescence.

In summary, we found that *T. delaisi* iris radiance is often visibly brighter to its own conspecifics than that of the substrate on which it lives. This effect can in part be attributed to red fluorescence, which increases the overall brightness, particularly when shaded.

## COMPETING INTERESTS

The authors declare that they have no competing interests.

## AUTHOR DETAILS

Animal Evolutionary Ecology, Institute for Evolution and Ecology, Department of Biology, Faculty of Science, University of Tuebingen, Auf der Morgenstelle 28, 72076 Tuebingen, Germany. This work was funded by a Reinhart Koselleck Project Grant Mi482/13‐1 from the Deutsche Forschungsgemeinschaft to N.K.M. P‐P.B. was supported by a Postdoctoral Fellowship by the Natural Sciences and Engineering Research Council of Canada.

## AUTHORS CONTRIBUTIONS

UKH and NKM designed the experiments and optimized the methodology. UKH, NKM, MGM, CMC, FW, and TG collected the data. UKH and PPB contributed in data analyses and drafting of the manuscript. MS and PPB implemented the visual modelling in visual modeling. UKH, NKM, PPB, MS, MGM, FW, TG, and CMC edited the manuscript. All authors read and approved the final manuscript.
