# Supplementary material for: Do the fluorescent red eyes of the marine fish Tripterygion delaisi stand out? In situ and in vivo measurements at two depths
Source: Ecol Evol. 2018 Apr 15;8(9):4685–94. doi: 10.1002/ece3.4025 (PMC5938470; doi:10.1002/ece3.4025)
Supplement: Supplementary file 1 [file ECE3-8-4685-s001.pdf]

Main substrates measured in -5 and -20 m

Family	Substrate (specific)	
Chlorophyceae	*Chaetomorpha linum*	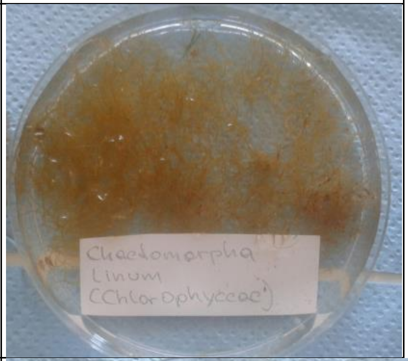
Phaeophyceae	*Cystoseira sp.*	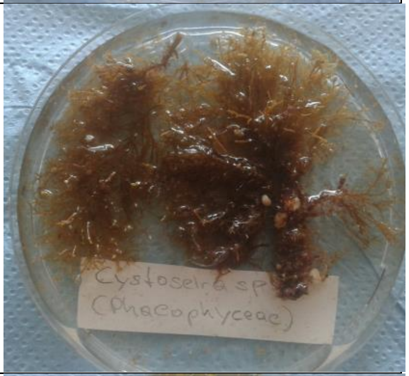
Phaeophyceae	*Dictyota linearis*	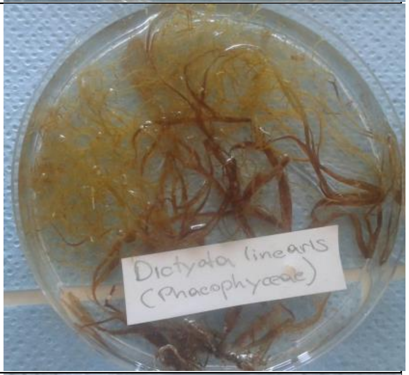
Chlorophyceae	*Halimeda tuna*	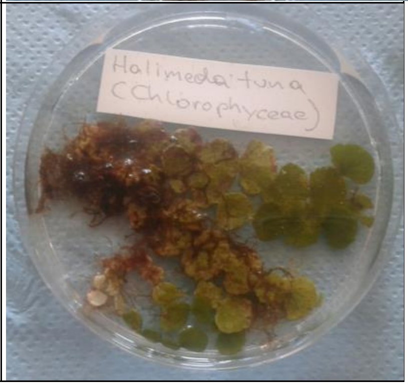

**Family**	**Substrate (specific)**	
Phaeophyceae	*Halopteris filiciana*	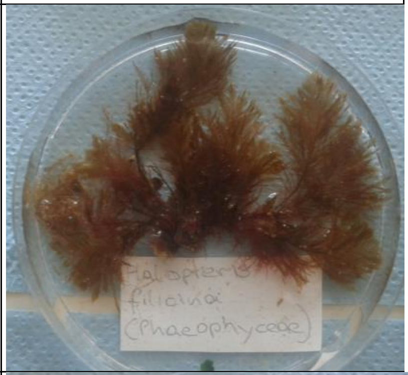
Rhodophyceae	*Jania rubens*	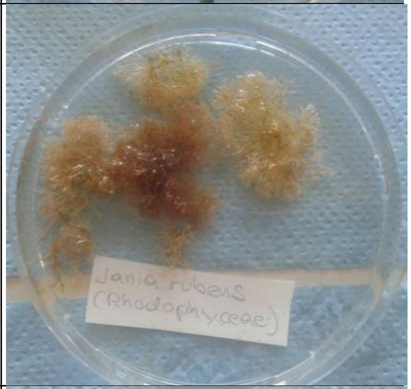
Phaeophyceae	*Padina pavonia*	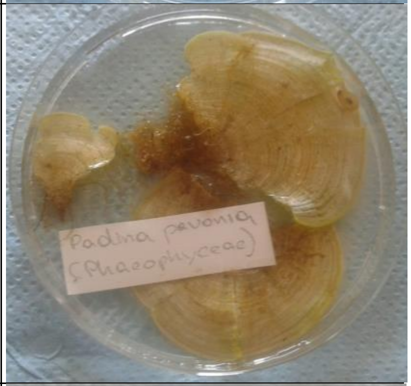
Chlorophyceae	*Udotea petiolata*	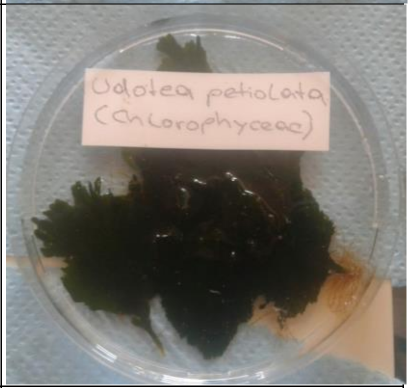
Chlorophyceae	Caulerpa racemosa	n.a.
Chlorophyceae	Codium bursa	n.a.
